# Extragustatory bitter taste receptors in head and neck health and disease

**DOI:** 10.1007/s00109-024-02490-0

**Published:** 2024-09-25

**Authors:** Jacob C. Harris, Robert J. Lee, Ryan M. Carey

**Affiliations:** 1 Department of Otorhinolaryngology, Perelman School of Medicine, University of Pennsylvania, Philadelphia, PA 19104 USA; 2 Department of Physiology, Perelman School of Medicine, University of Pennsylvania, Philadelphia, PA 19104 USA

**Keywords:** Bitter taste receptors, Innate immunity, Gustation, Genetics, Cancer

## Abstract

Taste receptors, first described for their gustatory functions within the oral cavity and oropharynx, are now known to be expressed in many organ systems. Even intraoral taste receptors regulate non-sensory pathways, and recent literature has connected bitter taste receptors to various states of health and disease. These extragustatory pathways involve previously unexplored, clinically relevant roles for taste signaling in areas including susceptibility to infection, antibiotic efficacy, and cancer outcomes. Among other physicians, otolaryngologists who manage head and neck diseases should be aware of this growing body of evidence and its relevance to their fields. In this review, we describe the role of extragustatory taste receptors in head and neck health and disease, highlighting recent advances, clinical implications, and directions for future investigation. Additionally, this review will discuss known *TAS2R* polymorphisms and the associated implications for clinical prognosis.

## Introduction

Of the five cardinal tastes, salty and sour tastes are detected by sodium- and acid-sensing ion channels respectively via type III taste bud cells [1]. Meanwhile, bitter, sweet, and umami ligands interact with G-protein coupled receptors (GPCRs) on taste bud cells. Sweet and umami tastes are regulated by taste family 1 receptor proteins (T1Rs), encoded by the *TAS1R1*, *TAS1R2,* and *TAS1R3* genes [2]. Bitter taste is recognized through taste family 2 receptor (T2R proteins encoded by *TAS2R* genes), for which 26 different GPCRs have been identified [3]. Bitter taste receptors have been identified both lingually and extralingually in organs including the upper and lower airway, GI tract, pancreas, and brain [4]. Gustatory bitter taste allows humans to recognize and avoid potentially toxic bitter compounds [5]. In extragustatory tissue, these calcium (Ca^2+^)-dependent pathways lead to tissue specific responses and can effect cell signaling, immunity, and carcinogenesis [6]. Until recently, the role of T2Rs in non-gustatory tissues has been poorly understood.

**Fig. 1:**
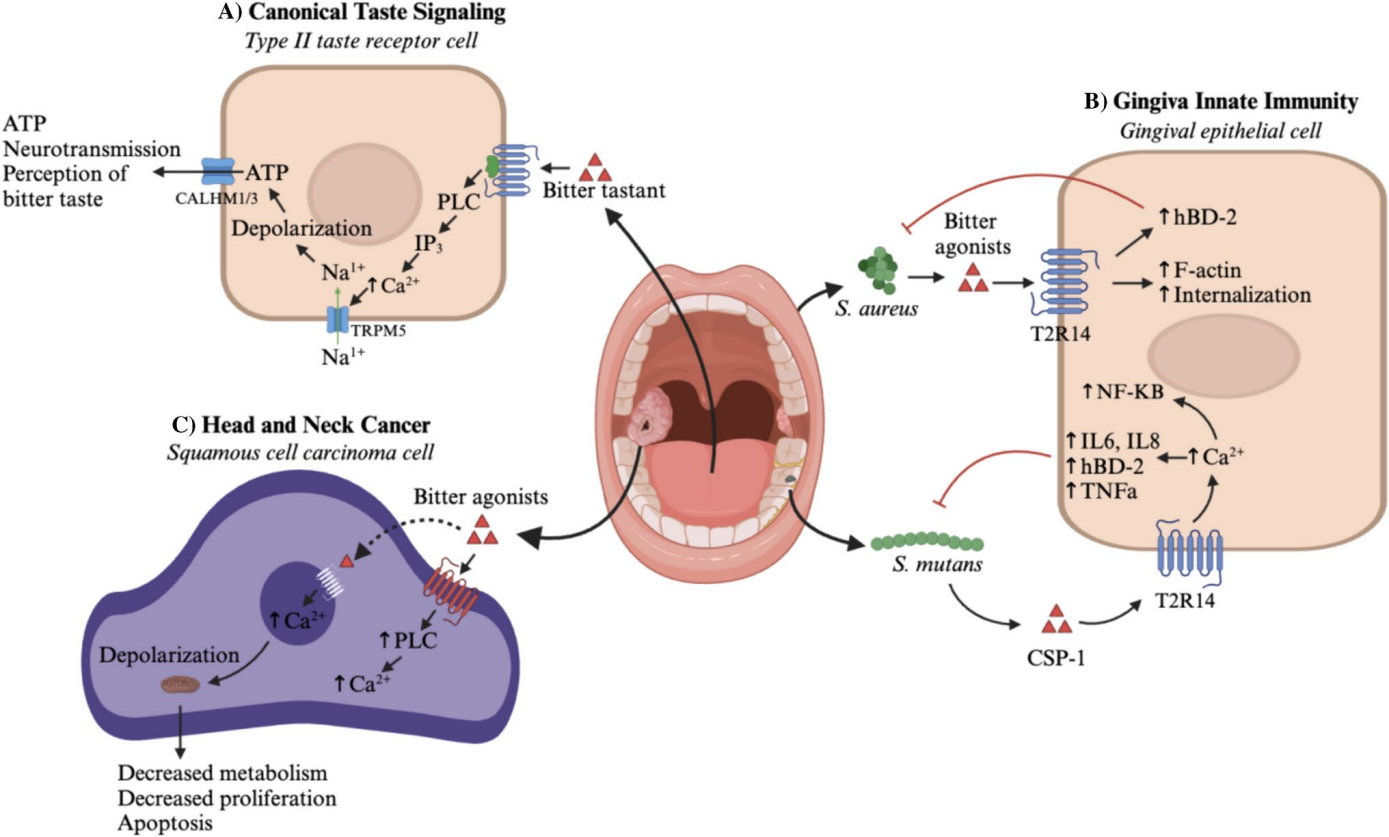
Bitter taste pathways within the oral cavity and oropharynx. (**A**) Canonical taste pathway. In the canonical gustatory bitter taste pathway, the bitter ligand binds a bitter taste receptor (T2R), leading to βγ activation of phospholipase C (PLC), generation of inositol-1,4,5-triphosphate (IP_3_), and release of intracellular Ca^2+^ from the endoplasmic reticulum [8]. The increased Ca^2+^ concentration induces opening of the TRPM5 sodium (Na^+^) channels, and Na^+^ influx triggers depolarization. The neurotransmitter ATP is released through CALHM1/3 channels into the synapse with the afferent taste axon, and the action potential is conveyed via the facial nerve to the gustatory cortex of the temporal lobe [9]. (**B**) Gingival innate immunity. Gingival epithelial cells (GECs) express T2Rs that mediate innate immune responses to gram-positive bacteria. Interactions between GEC T2R14 and *Staphylococci aureus* lead to the secretion of human B-defensin-2 (hBD2), as well as the upregulation of F-actin, which promotes cytoskeletal reorganization and bacterial internalization [35]. Meanwhile, the cariogenic pathogen *Streptococcus mutans* secretes competence stimulating peptides (CSPs). CSP-1 is an agonist for the T2R14 receptor, and activation leads to an intracellular Ca^2+^ surge and secretion of antimicrobial peptides including defensins and IL-8 [80]. (**C**) Head and neck cancer. Functionally active T2Rs are expressed by head and neck squamous cell carcinoma (HNSCC) tumor cells on the extracellular and nuclear membranes. Bitter agonists bind to GPCRs on both membranes, leading to Gα-gustducin activation, activation of PLC, and increased intracellular and intranuclear Ca^2+^ (Ca^2+^_nuc_). Increased Ca^2+^_nuc_ triggers mitochondrial depolarization, leading to decreased cellular metabolism, decreased proliferation, and apoptosis [99]. Figures created with BioRender

T2Rs on the tongue couple to heterotrimeric G proteins including Gα-gustducin (transducing 3, or GNAT3), while outside of taste cells, they couple to Gαi [7]. Activation of T2Rs by bitter ligands results in Gβγ activation of phospholipase C and generation of inositol-1,4,5-triphosphate (IP_3_) [8]. IP_3_ activates receptors on the endoplasmic reticulum (ER), leading to intracellular Ca^2+^ release. In the case of gustatory taste receptors, this results in depolarization and neurotransmission (Fig. 1A) [9]. Meanwhile, Gα-gustducin activates phosphodiesterases and Gαi inhibits adenylyl cyclases. Both processes reduce the concentration of cyclic-AMP (cAMP) and reduce protein kinase A (PKA) activity, which may be a negative regulator of type III IP_3_ receptor (IP_3_R3)–mediated Ca^2+^ release in taste cells [10]. While T2R cAMP decreases have been visualized in non-gustatory tissue, the role of these decreases is unknown [11].

Clinical research has demonstrated altered expression of T2R receptors in many infectious, autoimmune, and oncologic diseases, suggesting T2R-mediated disease processes [12, 13]. Importantly, significant genetic variability exists in human bitter taste receptors. In a well-documented example, the *TAS2R38* gene commonly carries single nucleotide polymorphisms (SNPs) resulting in measurable differences in taste recognition of phenylthiocarbamide (PTC) and 6-n-propylthiouracil (PROP) [14]. The more functional allele, characterized by the proline-alanine-valine sequence (PAV), commonly called the “supertaster” allele, is differentiated from the less functional, or “non-taster” allele, characterized by an alanine-valine-isoleucine sequence (AVI). Individuals with the PAV/PAV allele can be up to 50 times more sensitive PROP tasters [15]. Recent literature describes an emerging role for these genotypic variations in not only differential taste perception but in differing susceptibility to diseases and response to medical therapy among affected patients [13]. Within otolaryngology, bitter taste receptors have been most thoroughly studied for their role in innate immunity of the upper airway [13, 16]. However, T2Rs have also been linked to oral health and infection, otologic health, head and neck cancer, and thyroid function, among other systems and pathologies. In this review, we summarize the bitter taste receptor literature of interest to otolaryngologists, with an emphasis on possible clinical applications and areas for future investigation.

## Upper airway (sinonasal mucosal immunity)

### Expression of bitter taste receptors in the upper airway

The upper airway, consisting of the nasal cavity and paranasal sinuses, is constantly exposed to external pathogens [17]. Innate immunity in the sinonasal airway is comprised of synergistic systems including mucociliary clearance and the secretion of antimicrobial compounds. The mucociliary clearance system includes goblet cells, which secrete mucus, and motile respiratory cilia, which have a 9 + 2 microtubule structure and are highly expressed on the surface of airway epithelial cells [16]. These cilia beat continuously to move mucus and entrapped pathogens through the airway surface liquid (ASL) [18]. Meanwhile, antimicrobial peptides, reactive oxygen species, and reactive nitrogen species are produced by the airway epithelium and secreted into the ASL [16]. In these processes, both bitter and sweet taste receptors play important regulatory roles [19].

**Fig. 2:**
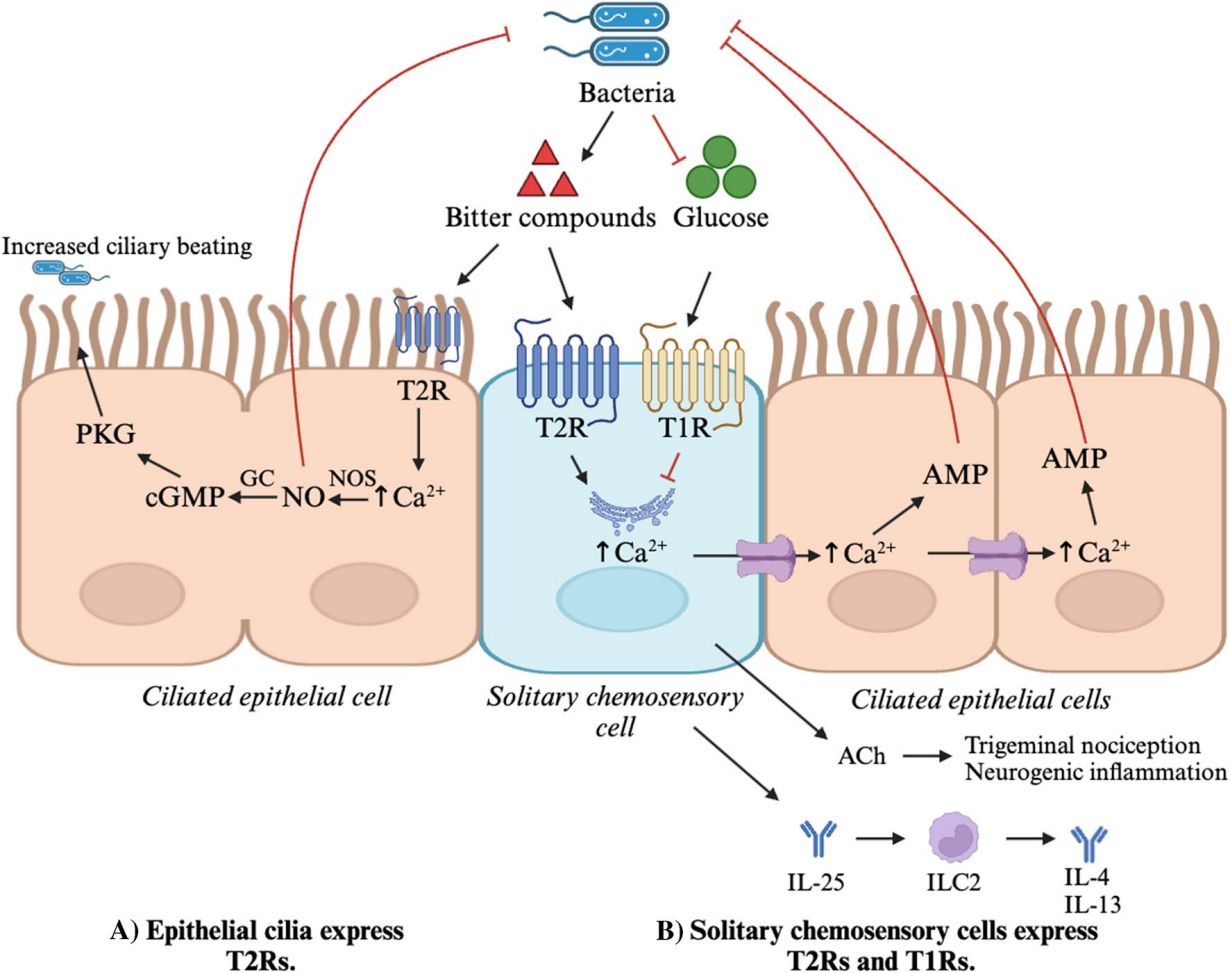
Type 2 bitter taste receptors (T2Rs) expressed by ciliated epithelial cells and by solitary chemosensory cells (SCCs) mediate innate immune functions in the sinonasal epithelium. (**A**) Gram-negative pathogens such as *Pseudomonas aeruginosa* produce bitter quorum-sensing molecules including acyl-homoserine lactones (AHL) [11, 25]. Respiratory cilia express T2Rs including T2R4, 14, 16, and 38 and activation by AHL leads to increased intracellular Ca^2+^ and activation of Ca^2+^-dependent nitric oxide synthase (NOS) [18, 20, 25]. NOS produces nitric oxide (NO), which diffuses across the cell membrane to exert direct antibacterial effects [24]. Within the respiratory epithelial cells, NO additionally activates guanylyl cyclase to produce cyclic guanosine monophosphate (cGMP), which activates protein kinase G (PKG) [20]. PKG phosphorylates ciliary proteins to increase the rate of ciliary beating and the velocity of mucociliary clearance [22]. (**B**) Solitary chemosensory cells (SCCs) express both bitter (T2Rs, specifically T2R10, 46, and 47) and sweet (T1Rs) receptors [18]. Bacterial bitter compounds bind and activate T2Rs inducing Ca^2+^ release from the endoplasmic reticulum (ER). Ca^2+^ spreads through gap junctions to neighboring ciliated epithelial cells and release stored antimicrobial proteins (AMPs) and peptides such as β-defensins and pro-inflammatory cytokines such as IL-25 [28–30]. SCCs release acetylcholine (ACh) triggering trigeminal nociceptors to induce neurogenic inflammation [32]. Negative feedback is exerted via T1Rs, which inhibit intracellular Ca^2+^ release in the presence of free glucose. Bacteria consume luminal glucose, reducing negative feedback and amplifying the T2R-mediated response [28, 39]

Within the sinonasal epithelium, bitter and sweet taste receptors are found on both ciliated epithelial cells and solitary chemosensory cells (SCCs) [20, 21]. Ciliated epithelial cells are the most predominant cell type in the sinonasal airway. T2Rs in the airway were first identified in bronchial ciliated cells [22]. While airway motile cilia were long thought to serve only a mechanical role in the transport of mucus, the presence of these taste receptors demonstrated chemosensory function. Sinonasal ciliated cells express multiple T2R isoforms (including T2R4, T2R14, T2R16, and T2R38) [16], and activation of these receptors by bitter ligands leads to Ca^2+^-dependent nitric oxide (NO) synthesis via the endothelial NO synthase (eNOS) enzyme [20]. Increased concentrations of intracellular NO activate soluble guanylyl cyclase to produce cyclic guanosine monophosphate (cGMP), activate protein kinase G (PKG), and increase the rate of epithelial ciliary beating, contributing to more rapid movement of mucus via the mucociliary clearance system [22, 23]. NO additionally diffuses across the cell membrane to directly exert antimicrobial effects [24]. These pathways act in response to infection with pathogens such as *Pseudomonas aeruginosa* [25] (Fig. 2A).

SCCs are rare cells also known as “tuft” or “brush” cells due to the presence of apical microvilli that may play a role in sensing the mucosal environment [26]. Nasal SCCs may have similarities to intestinal tuft cells that regulate type 2 immune responses in intestinal infections [27]. SCCs express unique T2R isoforms (T2R10, 46, and 47) compared to ciliated epithelial cells [18]. In parallel to the ciliated epithelial responses described above, SCCs respond to bitter agonism by stimulating a Ca^2+^ wave that passes through gap junctions to neighboring epithelial cells and causes rapid release of stored antimicrobial peptides (Fig. 2B) [28, 29]. SCCs also secrete inflammatory cytokines such as IL-25 which contribute to local inflammation in diseases such as chronic rhinosinusitis (CRS) [20, 30]. IL-25 is associated with type 2 (Th2) immunity and acts on group 2 innate lymphoid cells (ILC2s) to upregulate production of IL-4 and IL-13 [16, 30, 31]. In addition, Saunders et al*.* demonstrated that mouse sinonasal SCCs respond to bitter agonism by releasing acetylcholine (ACh) onto trigeminal nociceptors, activating neurogenic inflammatory pathways [32].

### Activation of T2Rs by bacterial products

Numerous bacterial products activate T2Rs. For instance, many gram-negative bacteria secrete quorum-sensing molecules (QSMs) as a form of intercellular communication [33]. Examples include acyl-homoserine lactones (AHLs) [25] and quinolones [11] produced by *P. aeruginosa* and *Escherichia coli*. These QSMs are bitter agonists of T2R38, and in the setting of infection, increasing ligand concentrations will trigger T2R-driven innate immune responses [25]. T2R38 is activated by other bacterial metabolites including acetone, t-butanone, 2-pentanon, 2-methylpopanal, dimethyl disulfide, methyl mercaptan, and γ-butyrolactone [34]. The importance of T2R38 agonism is demonstrated by higher rates of intracellular Ca^2+^ signaling, NO release, mucociliary transport velocity, and bactericidal activity in PAV/PAV individuals, and clinical data demonstrates that PAV/PAV individuals experience less frequent upper respiratory infections with gram-negative bacteria compared to patients with PAV/AVI or AVI/AVI *TAS2R38* genotypes [25].

Other T2Rs contribute to innate immunity. Competence stimulating peptides (CSPs), produced by gram-positive bacteria, are agonists of T2R14 [35]. Neutrophilic T2R138 detects the ligand AHL-12, allowing for intracellular processing and degradation of *P. aeruginosa* [36]. Denatonium benzoate activates eight T2Rs (but not T2R38) to induce a bactericidal response through release of β-defensins [29]. T2R-mediated NO production and increased ciliary beating are observed with unknown ligand(s) produced by *Staphylococcus aureus* and *Bacillus cereus* [37]. These pathways are analogous to the recognition of pathogen-associated molecular patterns (PAMPs) by receptors like toll-like receptors (TLRs) in classic innate immunity. However, the release of NO occurs more rapidly via T2Rs than with TLR activation, suggesting these two processes may complement each other as they occur on different time scales [16].

### The role of sweet and umami taste receptors in mucosal immunity

Sweet taste receptors, or T1R2/3 s, are coexpressed along with T2Rs in solitary chemosensory cells (SCCs) [38]. Activation of these T1R2/3 s reduces intracellular calcium signaling, and thus, sweet taste receptors act as a negative break on the taste receptor–mediated immune response. This negative regulation occurs at physiologic ASL glucose concentrations of 0.5–1 mM [28, 29]. In the setting of bacterial infection, increasing volumes of bacteria consume luminal glucose and decrease T1R2/3 activation [16]. This reduces negative feedback and unleashes a robust T2R-mediated response triggered by bitter bacterial ligands [28, 39]. In a physiologic state, sweet taste receptors may prevent an unchecked immune response against the commensal bacteria that colonize the sinonasal airway. However, dysfunction of this regulatory pathway can contribute to disease. For example, *Staphylococcus aureus* can produce D-amino acids (including D-Phe, D-Trp, and D-Leu) that activate T1R receptors and attenuate SCC intracellular Ca^2+^signaling [18, 40]. In addition, when luminal glucose levels are elevated, such as in diabetes or chronic rhinosinusitis, sinonasal immunity may be impaired [41, 42].

Recent work by McMahon et al*.* has identified umami T1R1/3 receptors in airway basal cells, where they detect amino acids (such as l-glutamate) and modify epithelial Ca^2+^ content. Modification of this pathway can limit Ca^2+^-linked caspase activation during apoptotic stimulation [43]. It is likely that other cell types in the epithelium (e.g., goblet cells, ionocytes) also express taste receptors that regulate important physiological functions, though many of these pathways have yet to be fully described.

### Clinical implications of upper airway taste receptors

A growing body of evidence associates sinonasal T2R expression with clinical outcomes. For example, chronic rhinosinusitis (CRS) is a highly prevalent disease often associated with chronic bacterial colonization and immune dysfunction [44]. As noted previously, the *TAS2R38* gene is polymorphic, and variations in *TAS2R38* expression may account for differing susceptibilities to diseases such as CRS [25]. Multiple studies show CRS to be more prevalent in patients with PAV/AVI and AVI/AVI genotypes compared to the PAV/PAV genotype [45–47]. At the time of sinus surgery, CRS patients with nonfunctional *TAS2R38* alleles are more likely to show active colonization by gram-positive [48] and gram-negative bacteria [25]. Similarly, CRS patients with AVI phenotypes are more likely to have medically incalcitrant disease, to require endoscopic sinus surgery [13, 49], and to report worse 6-month postoperative outcomes [50]. While CRS without nasal polyps was traditionally more strongly associated with *TAS2R38* expression [51], recent literature has demonstrated that *TAS2R38* is expressed in nasal polyps and that patients with advanced CRS with nasal polyps (CRSwNP) show decreased *TAS2R38* expression in their nasal mucosa compared to healthy controls [52]. Cantone et al. additionally observed that patients with CRSwNP with the AVI phenotype were more likely to present with biofilm formation at the time of surgery [47]. SCCs are upregulated in the mucosa of patients with fungal rhinosinusitis, emphasizing the role bitter taste plays in the pathogenesis of chronic nasal immunity and dysfunction [53].

The *TAS2R38* genotype may act as a disease modifier for cystic fibrosis (CF) [54]. An Italian cohort study found that the frequency of the PAV/PAV allele was significantly lower in CF patients requiring surgery for nasal polyps and for CF patients with childhood chronic pulmonary colonization by *P. aeruginosa* compared to controls [54]*.* Similarly, Piatti et al*.* reported that patients with primary ciliary dyskinesia (PCD) and the PAV/PAV genotype were less likely to experience frequent disease exacerbations or to be colonized with *P. aeruginosa* compared to the PAV/AVI or AVI/AVI genotypes [55]. Patients with CF and CRS with less functional *TAS2R38* alleles reported significantly worse rhinologic symptoms compared to PAV/PAV controls [56]. In CF patients, T2R signaling may occur at normal levels but results in decreased NO production compared to non-CF controls, which increase susceptibility to gram-negative infections [57]. *TAS2R38* expression is also correlated with susceptibility to viral infection. Barham et al*.* observed more frequent infections, hospitalizations, and increased duration of disease for coronavirus-2019 (COVID-19) patients with AVI/AVI genotypes [58, 59]. T2R38 sensitivity decreases with age, particularly for PAV/AVI heterozygotes [60], which might contribute to more severe COVID-19 disease in older cohorts [58]. Given these factors, taste receptor expression may have prognostic utility for clinicians [61]. For example, Taha et al*.* experimented with assigning treatment protocols for COVID-19 patients based on the negative prognostic implications of a “non-taster” T2R38 status [62]. In addition, gustatory taste tests may be useful for predicting severe or recalcitrant sinonasal disease, specifically CRS and associated disorders [63].

While few treatments for sinonasal disease explicitly target bitter taste receptors, many pharmaceuticals activate T2Rs. For example, fluoroquinolones and macrolides, common antibiotics for airway infections, are bitter agonists [64, 65]. Other, more experimental taste receptor therapies include flavones and alkaloids, as well as T1R antagonists. Kuek et al*.* demonstrated that topical application of the T2R agonist diphenhydramine increased ciliary beat frequency and NO production for CF and non-CF cell cultures, while reducing *P. aeruginosa* growth and biofilm production, suggesting potential clinical usefulness for CF-related CRS [66]. Flavones interact with T2R14, a receptor that closely localizes with T2R38, and have antibacterial and anti-inflammatory effects in patients with upper respiratory infections, particularly AVI/AVI patients [67]. Quinine, an agonist for several T2Rs, increases ciliary movement and NO production in vitro, and taste testing for quinine may be useful for identifying CRSwNP [68]. These compounds may be particularly useful because they do not interact with T2R38 and thus could play a role in the treatment of patients with deficiencies at this receptor [69]. As discussed above, T1Rs downregulate T2R-mediated pathways, and *Staphylococci* may exhibit immune escape due to their production of d-amino acids [28]. McMahon et al. used lactisole, a sweet/umami T1R3 antagonist, to amplify the effects of bitter denatonium benzoate on airway basal cells [70]. However, commensal bacterial production of D-amino acids may limit biofilm formation and prevent infection by more virulent species [71], limiting the current clinical use of T1R antagonists [69]. In summary, T2R expression and polymorphisms provide valuable prognostic data for a range of upper airway diseases, and recent literature suggests that endogenous T2R-mediated pathways could prove valuable pharmaceutical targets.

## Oral cavity

Although extragustatory taste receptors are sometimes referred to as “extra-oral,” this is a misnomer as taste receptors mediate both gustatory and non-gustatory pathways within the oral cavity. Gustatory oral taste receptors influence oral and dental health and disease primarily through dietary and behavioral influence [72, 73]. For instance, the *TAS1R2* gene (and specifically the rs35874116 SNP) is associated with increased dietary sugar intake and with increased risk of severe early childhood caries [74–76], while the *TAS2R38* gene is protective against dental carries [77]. In an online cross-section survey, those with greater self-reported taste intensity and stronger aversion to bitter flavors reported more frequent teeth brushing [78]. A genomic study in China demonstrated higher rates of the PAV/PAV *TAS2R38* genotype and subjective preference for bitter taste among moderate and heavy smokers [79]. While none of these studies imply causation, their data emphasize the contribution of gustatory taste to oral health and disease susceptibility.

Meanwhile, non-gustatory oral taste receptors contribute to innate immunity in the oral cavity in a manner analogous to the sinonasal epithelium (Fig. 1B**)**. Gingival epithelial cells (GECs) express T2Rs that respond to bitter ligands produced by both commensal and pathogenic bacteria [35]. In humans, the interaction between T2Rs and the oral microbiome was first observed for *Streptococcus mutans*, a gram-positive cariogenic bacterium that produces competence stimulating peptides (CSPs) [80]. CSP-1 acts as ligands for GEC T2R14, inducing Ca^2+^ signaling, upregulation of NF-κB, and the secretion of cytokines and chemokines including interleukin (IL)-6, IL-8, and tumor necrosis factor alpha (TNFα) [80]. T2R14 additionally mediates intraoral innate immunity against *S. aureus* through epithelial internalization of pathogens and the production of human beta defensin-2 (hBD2) [35]. T2R14 knockout decreases GEC internalization of *S. aureus* through the inhibition of F-actin-mediated cytoskeletal reorganization [35]. *TAS2R* expression may additionally regulate autophagy in the oral microenvironment [81]. The degree of T2R regulation varies genotypically; for example, the PAV/PAV allele is associated with greater GEC upregulation of T2R38 and greater production of hBD-2 after exposure to *S. mutans* than the AVI/PAV or AVI/AVI alleles [82]. In addition, research on human gingival fibroblasts has demonstrated T2R16 and T2R50 exert anti-inflammatory effects by downregulating IL-6 release in the presence of bacterial lipopolysaccharides [83, 84]. The authors demonstrated a linear relationship between the taste threshold of a bitter compound and the threshold for the anti-inflammatory pathway [83], highlighting the parallel effects of gustatory and extragustatory T2Rs.

These data and early experiments suggest there may be a clinical role for taste receptors in the treatment of oral disease. In mouse models, topical dental treatments with bitter compounds ameliorate periodontitis and alveolar bone loss in wild-type tasters, but not in mice without gustducin-expression [85, 86]. In human gingival fibroblasts, activation of T2R16 with salicin antagonizes NF-κB signaling and reduces the release of the inflammatory cytokines associated with chronic periodontitis [84]. Further research is needed to develop these findings into practical clinical applications.

## Middle ear

There are histologic similarities between the upper airway and middle ear. Krasteva et al. identified both bitter and umami receptors within the auditory tubes of mice [87], and in 2021, Kaufman et al. identified T2Rs in human middle ear and mastoid mucosa [88]. The relative expression of T2Rs in these regions is highly variable between patients, with T2R50 expressed most commonly [88]. The T2R50 receptor is not well understood; however, Kaufman linked genetic variations to middle ear disease. Patients with the rs137625 I allele of *TAS2R50* were observed to be at increased risk for COM, and conversely, patients with COM were found to have reduced bitter taste sensitivity independent of disease involvement of the chorda tympani [88]. To our knowledge, this is the only study exploring bitter taste reception within the middle ear, and future research should explore the role of T2Rs in mobilizing innate middle ear immunity against otopathogenic bacteria.

## Head and neck squamous cell carcinoma

Cancer cells express functional bitter taste receptors across a range of tumor pathologies, including gastrointestinal, breast, and pancreatic malignancies [89]. *TAS1R* and *TAS2R* expression have been linked to cancer risk and survival outcomes for multiple solid tumors [90]. Similarly, *TAS2R38* polymorphisms modify gastrointestinal and breast cancer risk [91–94]. T2R agonism leads to tumor cell apoptosis in breast and ovarian cancers [95–97], suggesting bitter taste receptors may offer both prognostic and therapeutic value. Emerging data suggests that T2Rs are important in head and neck squamous cell carcinoma (HNSCC), a highly prevalent disease with significant morbidity and mortality commonly treated by otolaryngologists [98].

HNSCC cells express various T2Rs (including TR4, 14, 19, 20, 30, 43, and 45) both on the cell and nuclear membranes [99]. Carey et al. demonstrated that HNSCC T2R activation induces an intranuclear Ca^2+^ surge and mitochondrial depolarization, leading to decreased cell metabolism, decreased proliferation, and apoptosis [99]. (Fig. 1C). In a review of The Cancer Genome Atlas, increased HNSCC expression of *TAS2R*s, and *TAS2R4* specifically, was associated with improved overall survival [99]. Although individual T2Rs have been linked to survival outcomes for several solid cancers [90, 100], further research is needed to determine whether specific T2Rs or *TAS2R* polymorphisms directly modify HNSCC risk and outcomes.

Bitter tasting compounds have been found to be cytotoxic to cancer cells in various organ systems [12, 100, 101] and to improve the efficacy of traditional chemotherapy by inhibiting cancer cell efflux transporters [100, 102]. There is now evidence of similar roles for targeted taste receptor therapies in head and neck cancer. Miller et al. demonstrated that lidocaine, a potent T2R14 agonist, inhibits both cell proliferation and the ubiquitin–proteasome system and induces apoptosis in HNSCC cells through Ca^2+^-dependent mechanisms [103]. Normal gingival keratinocytes expressed less T2R14 and were not killed with the same lidocaine exposure. These authors also suggest a potential therapeutic role for lidocaine in HPV-associated HNSCC, which highly expresses *TAS2R14* [103]. McMahon et al. demonstrated that bitter agonists triggered an elevation in nuclear Ca^2+^ and apoptosis in de-differentiated primary airway cells and airway cancer cells, but not in normally differentiated tissue [104]. Current treatments for HNSCC, including surgery, radiation, and chemo- and immunotherapies, are highly morbid, and head and neck oncologists are increasingly interested in targeted, less toxic therapies to improve patient quality of life [98, 105, 106]. In a rapidly changing field, bitter taste receptors may demonstrate prognostic, immunologic, and pharmaceutical potential for patients with head and neck cancer.

## Thyroid

In 2015, Clark et al. demonstrated that thyrocytes express T2Rs and that bitter taste receptor agonism decreased Ca^2+^ signaling and iodine efflux in a TSH-dependent process [107]. Polymorphisms in *TAS2R42* were found to be associated with differing levels of free serum T3 and T4 [107]. In an analysis of patients with and without thyroid disease, genetic variations in *TAS2R38* were associated with thyroid function and metabolism, and the presence of a PAV allele was protective against thyroid dysfunction (particularly hyperthyroidism) [108]. Conversely, patients with subclinical hypothyroidism were found on taste testing to have reduced sensitivity to bitter tastes with improvement after levothyroxine treatment [109]. A retrospective study of the Gene Expression Omnibus showed that, among other genes, *TAS2R16* and *TAS2R42* were differentially expressed in patients with and without Hashimoto’s thyroiditis [110]. In a case–control study of Korean patients, the *TAS2R3/4* CC haplotype was associated with reduced concentrations of triiodothyronine (T3) and was protective against papillary thyroid carcinoma [96]. Although the mechanisms of this observed effect are not fully understood, the authors hypothesized altered chemosensation of exogenous toxins and reduced stimulatory activity of T3 may provide anticarcinogenic protection [96]. As our understanding of T2Rs in thyroid tissue continues to grow, there may be opportunities for targeted endocrine and anticancer therapies. For example, propylthiouracil (PTU), used in the treatment of hyperthyroidism, is a known agonist of T2R4 and T2R36 [111], although further research is needed to understand what effect, if any, this agonism has on PTU’s clinical efficacy.

## Skin

Various T2Rs have been identified in epidermal keratinocytes, with expression differing by age, gender, and sun exposure [112]. These receptors have demonstrated chemosensory function [113], and various studies have suggested T2Rs contribute to the regulation of epidermal differentiation [114, 115], wound healing and aging [116], skin immunity [117], and hair follicle growth [118]. *TAS2R14* is expressed throughout the epidermis with measurable tastant-induced Ca^2+^ signaling [113]. These receptors may function to detect exogenous toxins and stimulate keratinocyte proliferation and epidermal repair [113]. In aged HaCaT cells, *TAS2R10* and *TAS2R16* overexpression was associated with more rapid wound healing on scratch-wound healing assays [116]. Bitter herbal compounds including melon [119] and teas [120] have demonstrated protective effects against photoaging. From an immunologic perspective, T2R38 is functionally expressed in skin-infiltrating lymphocytes and activation by bitter ligands decreased lymphocytic infiltration, possibly through inhibition of TARC-mediated migratory signaling [117]. Meanwhile, high rates of *TAS2R* mutations have been observed in cutaneous melanoma compared to other solid tumors [90]. Topical bitter compounds have been proposed as potential treatments for autoimmune dermatologic conditions including psoriasis and atopic eczema [114, 117, 121]. Otolaryngologists should be aware of the role bitter taste receptions appear to play in both medical and aesthetic dermatology.

## Nervous system

Bitter taste receptors are expressed within the central nervous system (CNS). Animal studies have identified T1Rs, Gα-gustducin, and T2Rs (specifically T2R4, T2R107, and T2R38) in murine neurons [122–124]. Zebrafish neurons express orphan T2Rs and responded to bitter compounds produced by *Streptococcus pneumoniae* with a Ca^2+^ surge and the secretion of antimicrobial cytokines [125]. Meanwhile, in humans, Kim et al*.* found *TAS2R14* to be highly expressed in neural tissue, with 100-fold greater expression in the cerebellum compared to lingual cells [126]. T2R14 is a highly promiscuous receptor and can interact with varied extra- and intracellular ligands, suggesting diverse functions [126]. While little research on *TAS2R* expression in neurologic disease is published, murine neuroblastoma cells induced to express *TAS2R8* and *TAS2R10* saw decreased expression of tumorigenic markers including hypoxia-inducible factor-1 alpha, decreased proliferation, and decreased tumor cell migration [89] and a human genome-wide association study found the rs667128 SNP on *TAS2R7* and the rs1033583 SNP on *TAS2R8* to be associated with glioma risk [127]. Together, these very early data suggest that bitter compounds may affect nervous system functions including immunity and tumorigenesis.

## Conclusion

Physiologically active extragustatory bitter taste receptors have been identified in nearly all organs of the head and neck, and their chemosensory and immunologic activity impacts health and pathology. *TAS2R* expression, and particularly the *TAS2R38* variants, has demonstrated prognostic value for sinonasal diseases, dental disease, otitis media, head and neck cancer, and thyroid cancer and dysfunction. Early research has shown that targeted treatments can be effective in vitro and ex vivo at amplifying native T2R-mediated immune pathways. Understanding bitter taste reception will be increasingly important for otolaryngologists as diagnostic and therapeutic uses of T2Rs become more widely studied and clinically accessible. T2R targeted therapies could emerge as a valuable adjunct to traditional antibiotic, chemotherapeutic, and surgical treatments of head and neck disease.

## Data Availability

Not applicable.
